# Supplementary material for: In situ microbeam surface X-ray scattering reveals alternating step kinetics during crystal growth
Source: Nat Commun. 2021 Mar 19;12:1721. doi: 10.1038/s41467-021-21927-5 (PMC7979818; doi:10.1038/s41467-021-21927-5)
Supplement: Supplementary file 1 — Supplementary Information [file 41467_2021_21927_MOESM1_ESM.pdf]

Supplementary Information for "*In Situ* Microbeam Surface X-ray Scattering Reveals Alternating Step Kinetics During Crystal Growth"

Guangxu Ju,[1,*] Dongwei Xu,[1,2] Carol Thompson,[3] Matthew J. Highland,[4] Jeffrey A. Eastman,[1] Weronika Walkosz,[5] Peter Zapol,[1] and G. Brian Stephenson[1,†]

[1]*Materials Science Division, Argonne National Laboratory, Lemont, IL 60439 USA*
[2]*School of Energy and Power Engineering, Huazhong University of Science and Technology, Wuhan, Hubei 430074, China*
[3]*Department of Physics, Northern Illinois University, DeKalb, IL 60115 USA*
[4]*X-ray Science Division, Argonne National Laboratory, Lemont, IL 60439 USA*
[5]*Department of Physics, Lake Forest College, Lake Forest, IL 60045 USA*

(Dated: February 15, 2021)

SUPPLEMENTARY METHODS 1: X-RAY SCATTERING

To characterize the behavior of A and B steps on a GaN single crystal (0001) surfaces, we performed *in situ* measurements of the CTRs during growth and evaporation in the OMVPE environment. For details on the GaN single crystal substrate, see vendor website (GANKIBAN^{TM} from SixPoint Materials, Inc., spmaterials.com). We used a chamber and goniometer at the Advanced Photon Source beamline 12ID-D which were designed for *in situ* surface X-ray scattering studies during growth[1]. For these experiments we used a modified chamber which had Be entrance and exit windows. A micron-scale X-ray beam illuminated a small surface area having a uniform step azimuth. To obtain sufficient signal, we used a wide-bandwidth "pink" beam setup similar to that described previously[2,3]. The beam incident on the sample had a typical intensity of 1.4×10^{12} photons per second at $E = 25.75$ keV, in a spot size of 10×10 μm. At the 2° incidence angle, this illuminated an area of 10×300 μm. X-ray scattering patterns were recorded using a photon counting area detector with a GaAs sensor having 512×512 pixels, 55 μm pixel size, located 1.1 m from the sample (Amsterdam Scientific Instruments LynX 1800). Slits downstream of the exit window block the scattering from the windows from reaching the region of the detector used to collect the CTR signal.

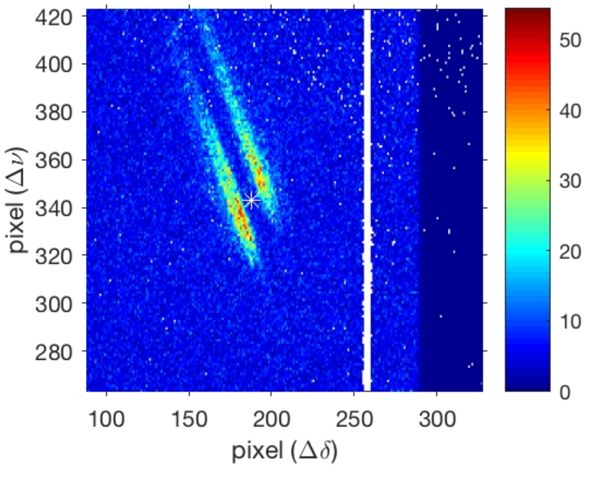

Supplementary Figure 1. Detector image from $(01\bar{1}L)$ scan for condition 4 (0.033 μmole min^{-1} TEGa, 0% H_2), showing intensity maxima from Ewald sphere cutting through CTRs from the $(01\bar{1}1)$ and $(01\bar{1}2)$ Bragg peaks. Position of central pixel, marked by star, is $(01\bar{1}L)$ with $L = 1.55$. Dark area on right is shadow of slits, and white vertical line is gap in pixels between detector chips. Pixels colored white are ignored due to excessive detector noise.

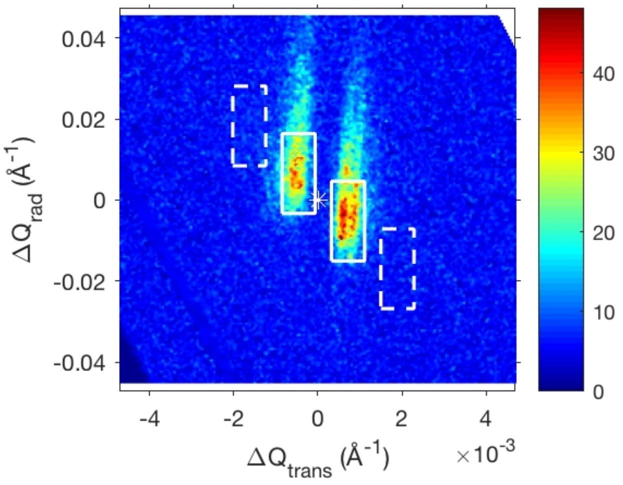

Supplementary Figure 2. Cut through reciprocal space at $L = Q_z c/2\pi = 1.55$, showing $(01\bar{1}1)$ and $(01\bar{1}2)$ CTRs. The in-plane Q_x and Q_y coordinates have been expressed as in-plane radial and transverse components ΔQ_{rad} and $\Delta Q_{\mathrm{trans}}$ relative to the central pixel at position $(01\bar{1}L)$. Solid and dashed rectangles show regions integrated to give CTR intensities and associated backgrounds, respectively.

To process the X-ray data from the area detector, raw images were first corrected for detector flatfield, eliminating pixels with excessive noise, and the signal was normalized to the incident intensity. Supplementary Fig. 1 shows a typical corrected detector image, with streaks from the $(01\bar{1}1)$ and $(01\bar{1}2)$ CTRs. Because of the $\sim 1\%$ energy bandwidth of the pink beam[3], the CTRs are broadened radially as well as being extended in the surface normal Q_z direction. To convert the images along an

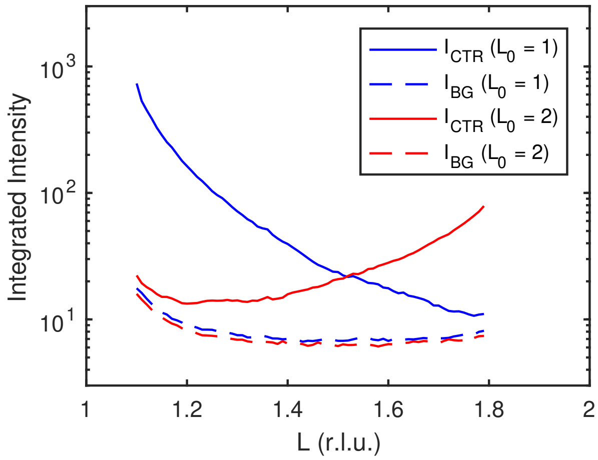

Supplementary Figure 3. Integrated total CTR and background intensities for the (01$\bar{1}$1) and (01$\bar{1}$2) CTRs, as a function of L between 1.1 and 1.8, for condition 4 (0.033 μmole min^{-1} TEGa, 0% H_2).

L scan to reciprocal space, the $Q_xQ_yQ_z$ coordinates of each pixel in each image were first calculated. The out-of-plane coordinate Q_z or L varies across each image, following the Ewald sphere. The in-plane coordinates Q_x and Q_y were converted to in-plane radial and transverse components $\Delta Q_{\rm rad}$ and $\Delta Q_{\rm trans}$ relative to the central position. The intensities and L values of each image were interpolated onto a fixed grid of $\Delta Q_{\rm rad}$ and $\Delta Q_{\rm trans}$. We then interpolated the sequence of intensities from the scan at each $\Delta Q_{\rm rad}$ and $\Delta Q_{\rm trans}$ onto a grid of fixed L values.

Supplementary Fig. 2 shows a typical cut through reciprocal space at fixed L. The peaks from the (01$\bar{1}$1) and (01$\bar{1}$2) CTRs are conveniently separated in $\Delta Q_{\rm trans}$ because of the 5° deviation of the step azimuth from [01$\bar{1}$0]; if the deviation had been zero, the peaks would have overlapped at $\Delta Q_{\rm trans} = 0$ because of the broadening in $\Delta Q_{\rm rad}$. Regions of $\Delta Q_{\rm trans}$ and $\Delta Q_{\rm rad}$ surrounding each CTR were defined to integrate the total intensity, with positions that vary with L to follow the CTRs. Likewise adjacent regions were defined to integrate an equivalent volume of background scattering. Such regions are shown as solid and dashed rectangles, respectively, in Supplementary Fig. 2. Supplementary Fig. 3 shows the mean total CTR intensities and backgrounds in these regions as a function of L for the scan between $L = 1.1$ and $L = 1.8$ for condition 4. The net CTR intensity was calculated by subtracting the background from the total for that CTR. We ran scans from $L = 0.4$ to $L = 0.9$, $L = 1.1$ to $L = 1.8$, and $L = 2.15$ to $L = 2.6$ on the (01$\bar{1}L$) and (10$\bar{1}L$) CTRs, skipping over the Bragg peaks to avoiding having the high intensity strike the detector. The L range covered on each CTR varied depending upon the region covered by the detector in reciprocal space during the scan.

In order to determine whether exposure to the X-ray beam was affecting the OMVPE growth process, we periodically scanned the sample position while monitoring the CTR intensity. For the conditions reported here, there was no indication that the spot which had been illuminated differed in any way from the neighboring regions. During growth at higher temperatures (e.g. 1250 K), we did observe local effects of the X-ray beam on the surface morphology.

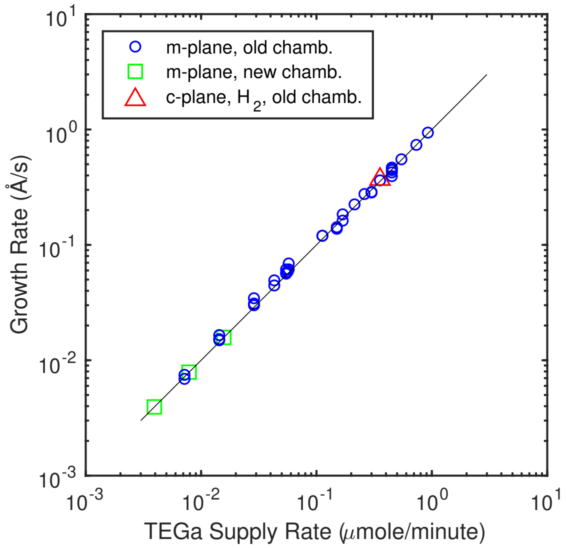

Supplementary Figure 4. Growth rate as a function of TEGa supply determined from CTR oscillations during layer-by-layer growth. Line is fit to new chamber data giving a deposition efficiency of 1.0 (Å s^{-1})/(μmole min^{-1}).

SUPPLEMENTARY METHODS 2: NET GROWTH RATES

Under the OMVPE conditions used, we observe that deposition of GaN is Ga transport limited (i.e. the deposition rate is proportional to the TEGa supply rate, nearly independent of T and NH_3 supply), and the net growth rate has a negative offset at zero TEGa supply corresponding to an evaporation rate that depends on T and the carrier gas composition (e.g. presence or absence of H_2). To determine the deposition rate for the conditions used in the X-ray study, we used the deposition efficiency (deposition rate per TEGa supply rate) determined from previous studies of CTR oscillations during layer-by-layer growth[3,4]. We also measured the evaporation rates at two higher temperatures and both carrier gas compositions (0% and 50% H_2), and extrapolated them to the lower temperatures studied here.

Supplementary Fig. 4 shows the growth rates measured from CTR oscillations during layer-by-layer growth as a function of TEGa supply[3,4]. In all cases the chamber flows were the same as in the X-ray study reported here (e.g. 2.7 slpm NH_3, 267 mbar total pressure). Almost all data points are for growth on m-plane (10$\bar{1}$0) GaN in N_2 carrier gas (0% H_2), which exhibits layer-by-layer mode

over a wide range of conditions. Data are shown from both a previous growth chamber ("old" chamber)[5] and the current growth chamber ("new" chamber)[1,3]. The two chambers were designed to have the same flow geometry, and the growth behavior of both appear to be identical. The data points from the previous chamber range in temperature from 848 K to 1064 K; the data points for the current chamber are for 867 K. The line shown is a fit to the data from the current chamber, which gives a deposition efficiency of 1.0 (Å s^{-1})/(μmole min^{-1}). One data point is shown for growth on c-plane (0001) GaN in 50% N_2 + 50% H_2 carrier gas at 900 K; layer-by-layer growth was only observed on (0001) GaN under this condition. It agrees with the m-plane data obtained in 0% H_2 carrier, suggesting that the same deposition efficiency can be used for (0001) GaN in either 0% or 50% H_2 carrier gas. We expect that there is negligible evaporation at 900 K in either carrier gas.

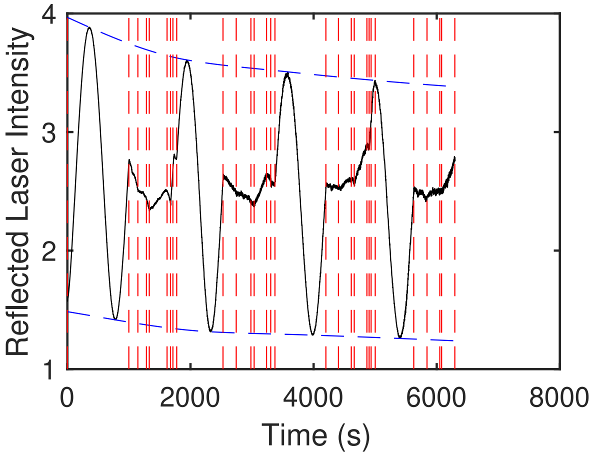

Supplementary Figure 5. Reflected laser signal during growth under various conditions. Vertical dashed lines show times at which conditions changed.

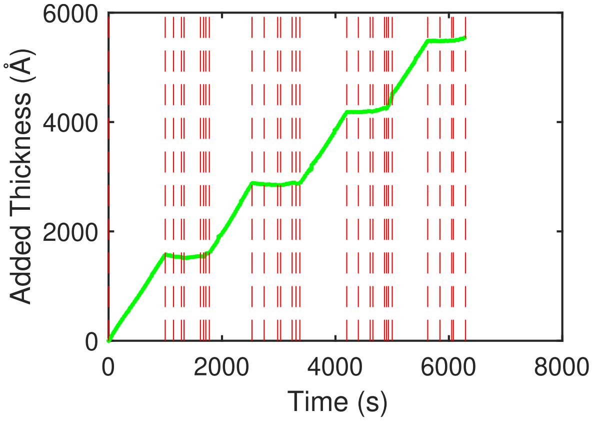

Supplementary Figure 6. Added thickness during growth under various conditions. Vertical dashed lines show times at which conditions changed.

To estimate the evaporation rate at the temperatures used in the X-ray study presented here (e.g. 1080 K), we used laser interferometry to observe the change in thickness of an (0001) GaN film on a sapphire substrate[1,6], under conditions of zero TEGa flow at higher T. As the film thickness $d(t)$ changes during growth or evaporation, the back-scattered laser intensity $I(t)$ oscillates with time t due to interference between light reflected from the film surface and the substrate/film interface, according to

$$I(t) - I_{\min} = [I_{\max} - I_{\min}] \left(\frac{1 + \cos[2\pi d(t)/d_0]}{2} \right), \quad (1)$$

where $I_{\min}(t)$ and $I_{\max}(t)$ are the envelope of the minima and maxima, which can vary with time as film roughness changes, and the thickness oscillation period is $d_0 = \lambda/2n$, where $\lambda = 6330$ Å is the wavelength of the light and n is the refractive index of GaN. This can be inverted to obtain the thickness evolution as

$$d(t) = \left(\frac{d_0}{2\pi} \right) \cos^{-1} \left(\frac{2[I(t) - I_{\min}(t)]}{I_{\max}(t) - I_{\min}(t)} - 1 \right). \quad (2)$$

Supplementary Fig. 5 shows the evolution of the laser signal with time during the experiment. We began by growing a full oscillation at a high growth rate to obtain initial values for $I_{\min}$ and $I_{\max}$. Once the signal had reach a value intermediate between these limits, where the phase of the oscillation is most accurately determined, we changed the TEGa flow f_{TEGa} to observe the net growth or evaporation rate at some fixed values of f_{TEGa}. Then we changed T and/or the carrier gas concentration, and repeated the process starting with growing a full oscillation at a high rate. The blue dashed curves in Supplementary Fig. 5 show the interpolated $I_{\min}(t)$ and $I_{\max}(t)$ envelopes. Supplementary Fig. 6 shows the thickness change with time extracted with Supplementary Eq. (2), using a value of $d_0 = 1302$ Å corresponding to $n = 2.431$[7,8]. Supplementary Fig. 7 shows expanded regions of the thickness evolution, where we varied f_{TEGa} for specific T and H_2 fractions. The solid lines show linear fits to extract the net growth rate in Å s^{-1} at each value of f_{TEGa}. This is equal to $Gc/2$, where G is the growth rate in ML s^{-1} and $c/2$ is the thickness of a monolayer (ML).

The extracted values of $Gc/2$ are given in Supplementary Table I and plotted in Supplementary Fig. 8 as a function of f_{TEGa}. We observe that $Gc/2$ becomes negative at $f_{\mathrm{TEGa}} = 0$ due to evaporation, and that evaporation is more rapid at higher T and when H_2 is present in the carrier gas. These evaporation rates in 50% H_2 are consistent with the rate of $\rho_0 G = -4.2 \times 10^{18}$ $m^{-2}s^{-1}$ or $Gc/2 = -0.97$ Å s^{-1} given in the literature[9] at a higher $T = 1300$ K with H_2 and NH_3 at a total pressure of 267 mbar. Also given in Supplementary Table I are the deposition efficiencies $d(Gc/2)/df_{\mathrm{TEGa}}$ obtained from linear fits to $Gc/2$ at the four values of f_{TEGa} for each T and H_2 fraction. The values are all similar to but slightly higher than the value of $d(Gc/2)/df_{\mathrm{TEGa}} =$ 1.0 (Å s^{-1})/(μmole min^{-1}) that we have observed from

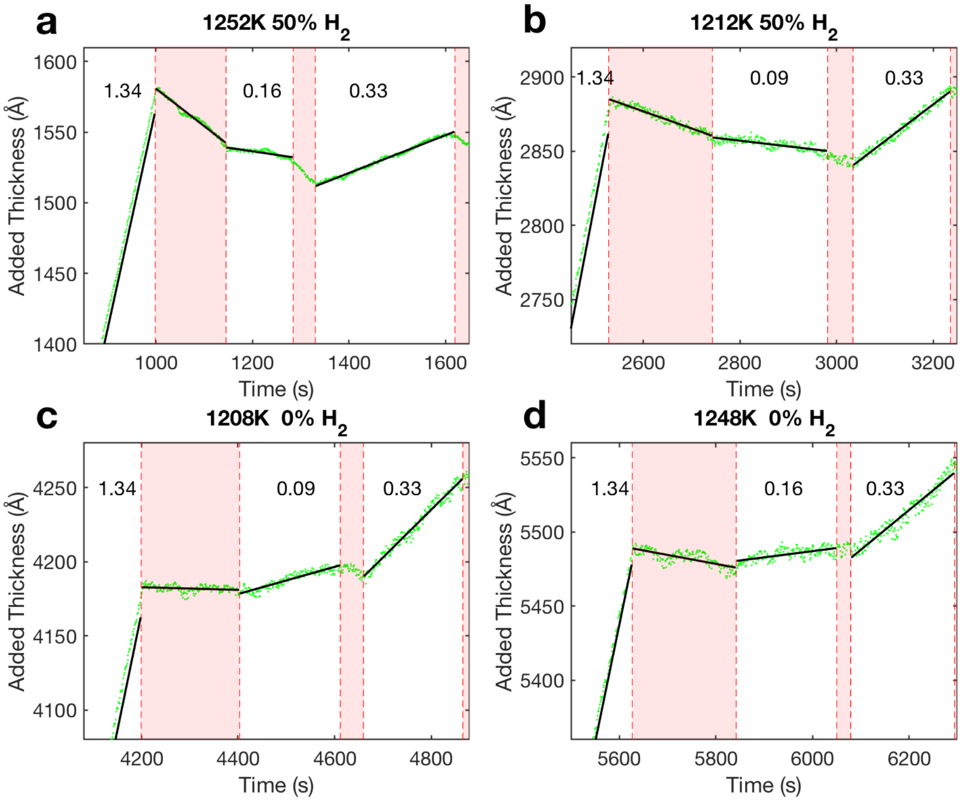

Supplementary Figure 7. Added thickness during growth under TEGa flows shown (μmole min^{-1}) **a**. at T = 1252 K, 50% H_2; **b**. at T = 1212 K, 50% H_2; **c**. at T = 1208 K, 0% H_2; **d**. at T = 1248 K, 0% H_2. Vertical dashed lines show times at which conditions changed. Black lines show fits to extract net growth rates. TEGa flow was 0 μmole min^{-1} for the shaded regions.

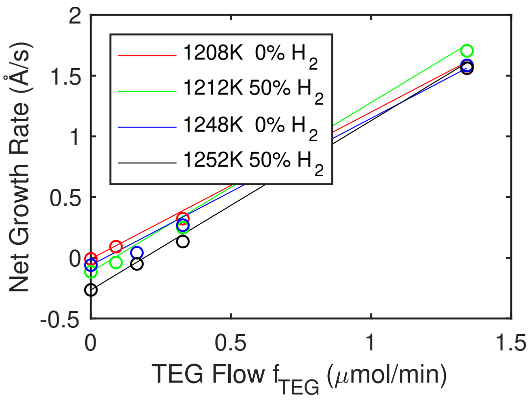

Supplementary Figure 8. Symbols show extracted net growth rates vs. TEGa flow for various T and carrier gas compositions.

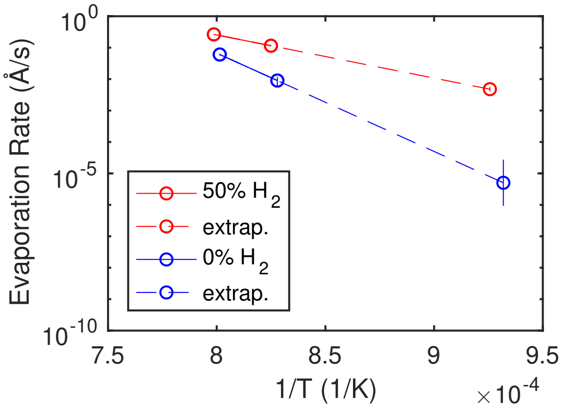

Supplementary Figure 9. Evaporation rate at zero TEGa flow as a function of T, with and without H_2, with extrapolation to lower T.

growth oscillations during layer-by-layer growth at lower T, described above[3,4]. The efficiency seems to be slightly larger for 50% H_2 compared with 0% H_2. This may indicate that the deposition efficiency can vary somewhat as the flow and diffusion fields vary in the chamber with T or carrier gas composition.

To obtain the evaporation rate at the lower T used in the X-ray experiments, we extrapolated the values at $f_{\mathrm{TEGa}} = 0$ for 50% H_2 or 0% H_2 assuming Arrhenius behavior of the evaporation rate, as shown in Supplementary Fig. 9. The fitted activation energies are 2.7±0.1 and 6.2 ± 1.2 eV in 50% and 0% H_2, respectively. We obtain evaporation rates of $4.8 \pm 0.8 \times 10^{-3}$ Å s^{-1} at $T = 1080$ K with 50% H_2, and 5×10^{-6} Å s^{-1} (with error limits of a

Supplementary Table I. Values of net growth rate $Gc/2$ extracted from laser interferometry measurements. We list the values for two temperatures and for carrier gas with and without H_2, as a function of TEGa flow f_{TEGa}. Also shown is fitted $d(Gc/2)/df_{\mathrm{TEGa}}$ for each T and carrier gas.

T (K)	H_2 in carr.	f_{TEGa} (μmole min^{-1})	$Gc/2$ (Å s^{-1})	$d(Gc/2)/df_{\mathrm{TEGa}}$ (Å s^{-1})/ (μmole min^{-1})
1208	0%	0.00	-0.009 ± 0.003	1.19 ± 0.03
		0.09	0.092 ± 0.003	
		0.33	0.322 ± 0.004	
		1.34	1.582 ± 0.002	
1212	50%	0.00	-0.115 ± 0.002	1.38 ± 0.05
		0.09	-0.038 ± 0.002	
		0.33	0.248 ± 0.003	
		1.34	1.705 ± 0.002	
1248	0%	0.00	-0.061 ± 0.004	1.27 ± 0.05
		0.16	0.042 ± 0.004	
		0.33	0.268 ± 0.005	
		1.34	1.584 ± 0.002	
1252	50%	0.00	-0.265 ± 0.004	1.40 ± 0.03
		0.16	-0.050 ± 0.003	
		0.33	0.134 ± 0.001	
		1.34	1.562 ± 0.001	

factor of 5) at $T = 1073$ K with 0% H_2. We have used these evaporation rates, as well as the low-temperature deposition efficiency of 1.0 (Å s^{-1})/(μmole min^{-1}) and the TEGa flow rates of 0 or 0.033 μmole min^{-1}, to calculate the net growth rates given in Table I in the main text for the 4 conditions studied.

SUPPLEMENTARY DISCUSSION 1: CHEMICAL POTENTIALS IN OMVPE

To calculate the CTR intensities to fit to the experimental profiles, we need to include the effect of reconstruction of the surface. The relaxed atomic structures and free energies of various surface reconstructions for GaN (0001) in the OMVPE environment containing NH_3 and H_2 have been calculated[10,11], leading to a phase diagram that can be expressed in terms of the chemical potentials of Ga and NH_3[11,12]. In this section we estimate these chemical potentials from the conditions in our experiments, to locate the appropriate region of the phase diagram and identify the predicted reconstructions in this region.

Supplementary Fig. 10 shows the predicted surface phase diagram[11]. The vertical axis is the chemical potential of NH_3 relative to its value at $T = 0$ K. This can be expressed as

$$\begin{aligned}\Delta\mu_{\mathrm{NH_3}}(T) &\equiv \mu_{\mathrm{NH_3}}(T) - \mu_{\mathrm{NH_3}}(0)\\ &= G^\circ_{\mathrm{NH_3}}(T) - G^\circ_{\mathrm{NH_3}}(0) + kT\log p_{\mathrm{NH_3}}, \quad (3)\end{aligned}$$

where $G^\circ_{\mathrm{NH_3}}$ is the free energy of NH_3 gas at a pressure of 1 bar obtained from thermochemical tables[13], and $p_{\mathrm{NH_3}}$

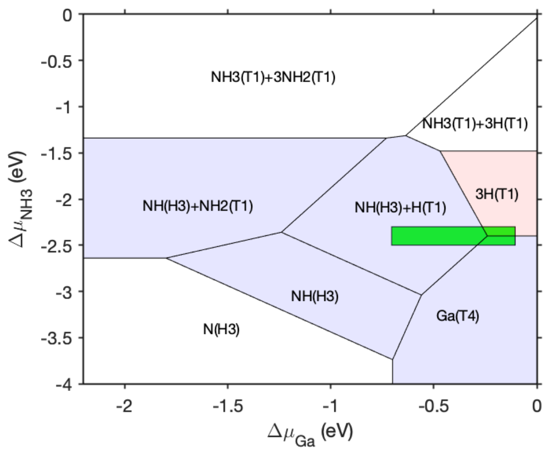

Supplementary Figure 10. Surface reconstruction phase diagram for GaN (0001), calculated in[11]. Green rectangle shows estimated position of our experimental conditions. Five shaded reconstructions near these conditions were considered in fits shown in Table II. The 3H(T1) reconstruction gives the best fit to all conditions.

is the partial pressure of NH_3 in the experiment. These can be evaluated at the experimental conditions. For $T = 1073$ K, the tables give $G^\circ_{\mathrm{NH_3}}(T) - G^\circ_{\mathrm{NH_3}}(0) = -2.1$ eV. Thus for $p_{\mathrm{NH_3}} = 0.04$ bar, one obtains $\Delta\mu_{\mathrm{NH_3}}(T) = -2.4$ eV.

The horizontal axis in Supplementary Fig. 10 is the chemical potential of Ga relative elemental liquid Ga. This can be related to the activity of N_2 using

$$\begin{aligned}\Delta\mu_{\mathrm{Ga}} &\equiv \mu_{\mathrm{Ga}}(T) - \mu^{\mathrm{liq}}_{\mathrm{Ga}}(T)\\ &= \Delta G^{\mathrm{GaN}}_f(T) - 0.5kT\log a_{\mathrm{N_2}}, \quad (4)\end{aligned}$$

where $\Delta G^{\mathrm{GaN}}_f$ is the free energy of formation of GaN from liquid Ga and N_2 gas at 1 bar, and $a_{\mathrm{N_2}}$ is the activity (effective partial pressure) of N_2.

In OMVPE, a chemically active precursor such as ammonia is typically used to provide the high nitrogen activity required to grow group III nitrides. The need for this can be seen in Supplementary Fig. 11, which shows the free energies of the reactions to form GaN and InN from the condensed metallic elements and either vapor N_2 or vapor NH_3 at 1 bar[13,14]. At typical temperatures used for growth of high quality single crystal films at high rates (e.g. 1000 K for InN, 1300 K for GaN), the formation energy from N_2 is positive, indicating that the nitride is not stable and cannot be grown from N_2 at 1 bar. In contrast, the formation energies of the nitrides (plus H_2 at 1 bar) from the metals and NH_3 are negative at all relevant growth temperatures, indicating that growth from 1 bar of NH_3 is possible.

However, actual OMVPE conditions do not correspond with equilibrium, because the very high partial pressures of N_2 and/or H_2 that would correspond to equilibrium with NH_3 at these temperatures are not allowed to accumulate. Thus, while formation of InN and GaN from

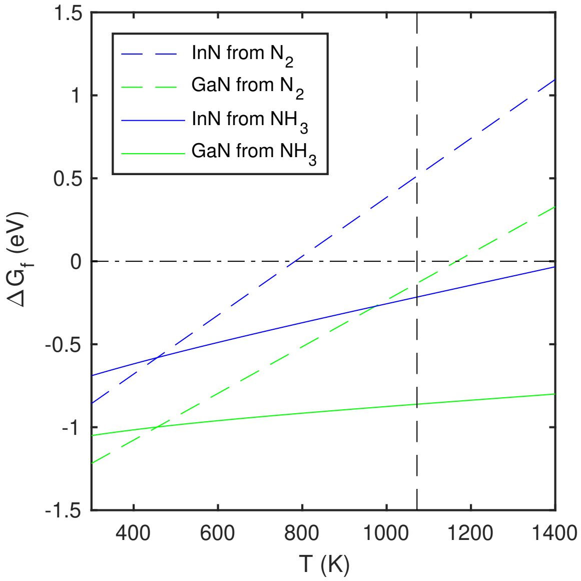

Supplementary Figure 11. Free energy of formation as a function of temperature of InN and GaN from the liquid metals and either vapor N_2 or NH_3 at 1 bar[13,14]. In the case of NH_3, this includes formation of H_2 at 1 bar. The vertical dashed black line corresponds to the T used in the current experiment.

NH_3 is energetically favored under OMVPE conditions, decomposition of these nitrides into N_2 is also energetically favored. This metastability is manifested in the oscillatory growth and decomposition of InN that has been observed[15]. Thus the kinetics of the reaction steps that determine the nitrogen activity at the growth surface are critical to understanding and controlling OMPVE growth of metastable nitrides.

In previous work we have measured the trimethylindium (TMI) partial pressures required to condense InN and elemental In onto GaN (0001)[15]. They can be analyzed to give experimentally determined values for the effective surface nitrogen activity arising from NH_3 under OMVPE conditions. The experiments were carried out using a very similar growth chamber[5] as that used for the *in situ* X-ray studies described below, using the same a total pressure of 0.267 bar, and the same NH_3 and carrier flows (2.7 standard liters per minute (slpm) NH_3 and 1.1 slpm N_2 in the group V channel, 0.9 slpm N_2 carrier gas for TMI in the group III channel). We have performed chamber flow modeling to calculate the equivalent TMI and NH_3 partial pressures p_{TMI} and $p_{\mathrm{NH_3}}$ above the center of the substrate surface as a function of inlet flows. At typical growth temperatures, an inlet flow of 0.184 μmole min^{-1} TMI corresponds to $p_{\mathrm{TMI}} = 1.22 \times 10^{-6}$ bar, and an inlet flow of 2.7 slpm NH_3 corresponds to $p_{\mathrm{NH_3}} = 0.040$ bar.

Supplementary Fig. 12 shows the p_{TMI}-T boundaries determined by *in situ* X-ray fluorescence and diffraction measurements for initial condensation of elemental In liquid or crystalline InN onto a GaN (0001) surface at

Supplementary Table II. Evaluation of N_2 activity and $\Delta\mu_{\mathrm{Ga}}$ at the GaN surface under OMVPE conditions. Formation energies of GaN and InN are from elements at standard conditions. TMI pressures at InN and In condensation boundaries are for $p_{\mathrm{NH_3}} = 0.04$ bar. Calculated $a_{\mathrm{N_2}}$ and $\Delta\mu_{\mathrm{Ga}}$ are thus also for $p_{\mathrm{NH_3}} = 0.04$ bar.

Quantity	Value as $f(T)$ (K) (eV)
$\Delta G_f^{\mathrm{GaN}}$ [14]	$-1.64 + 1.41 \times 10^{-3}T$
$\Delta G_f^{\mathrm{InN}}$ [14]	$-1.39 + 1.78 \times 10^{-3}T$
$kT \log p_{\mathrm{TMI}}^{\mathrm{InN}}$ [15]	$-1.309 + 0.88 \times 10^{-3}T$
$kT \log p_{\mathrm{TMI}}^{\mathrm{In}}$ [15]	$-3.843 + 3.47 \times 10^{-3}T$
$kT \log a_{\mathrm{In}}$ $= kT \log p_{\mathrm{TMI}}^{\mathrm{InN}} - kT \log p_{\mathrm{TMI}}^{\mathrm{In}}$	$-2.534 + 2.59 \times 10^{-3}T$
$kT \log a_{\mathrm{N_2}}$ $= 2(\Delta G_f^{\mathrm{InN}} - kT \log a_{\mathrm{In}})$	$2.288 - 1.63 \times 10^{-3}T$
$\Delta\mu_{\mathrm{Ga}}$ $= \Delta G_f^{\mathrm{GaN}} - 0.5kT \log a_{\mathrm{N_2}}$	$-2.784 + 2.225 \times 10^{-3}T$

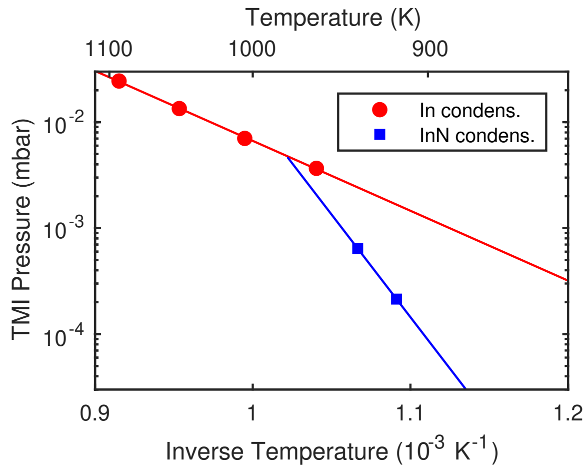

Supplementary Figure 12. Observed phase boundaries for condensation onto GaN (0001) of relaxed epitaxial InN (blue squares) and liquid elemental In (red circles) at $p_{\mathrm{NH_3}} =$ 0.040 bar[15].

$p_{\mathrm{NH_3}} = 0.040$ bar[15]. At TMI partial pressures above the boundaries shown, the condensed phases nucleate and grow on the surface; at lower p_{TMI}, the condensed phases evaporate. The InN and In condensation boundaries intersect at 979 K.

A relationship between the nitrogen and indium activities at the InN condensation boundary can be obtained from the equilibrium

$$\mathrm{In}_{vap} + \frac{1}{2}\mathrm{N}_2 \leftrightarrow \mathrm{InN}_{sol}, \quad (5)$$

which gives the chemical potential μ_i expression

$$\mu_{\mathrm{In}} + \frac{1}{2}\mu_{\mathrm{N_2}} = \mu_{\mathrm{InN}}, \quad (6)$$

and the activity a_i expression

$$kT \log a_{\mathrm{In}} + \frac{1}{2} kT \log a_{\mathrm{N_2}} = \Delta G_f^{\mathrm{InN}}(T), \tag{7}$$

where $\Delta G_f^{\mathrm{InN}}(T)$ is the formation energy of InN from liquid In and N_2 at 1 bar shown in Supplementary Fig. 12. We assume that the activity of In relative to liquid In at the InN boundary is equal to the ratio $a_{\mathrm{In}} = p_{\mathrm{TMI}}^{\mathrm{InN}}/p_{\mathrm{TMI}}^{\mathrm{In}}$, giving

$$kT \log a_{\mathrm{In}} = kT \log p_{\mathrm{TMI}}^{\mathrm{InN}} - kT \log p_{\mathrm{TMI}}^{\mathrm{In}} \tag{8}$$

at the experimental condition, $p_{\mathrm{NH_3}} = 0.040$ bar. Supplementary Eq. (7) can then be used to obtain the nitrogen activity relative to 1 bar (i.e. effective partial pressure of N_2 in bar) for $p_{\mathrm{NH_3}} = 0.040$ bar.

Supplementary Table II summarizes the calculations to obtain the nitrogen activity and $\Delta\mu_{\mathrm{Ga}}$ under our OMVPE conditions. The value of $kT \log a_{\mathrm{N_2}} = 0.55$ eV at the experimental temperature $T = 1073$ K gives the horizontal coordinate on the phase diagram from Supplementary Eq. (4) as $\Delta\mu_{\mathrm{Ga}} = -0.40$ eV. The value of $kT \log p_{\mathrm{NH_3}} = -0.30$ eV at the experimental temperature $T = 1073$ K gives the vertical coordinate on the phase diagram from Supplementary Eq. (3) as $\Delta\mu_{\mathrm{NH_3}} = -2.40$ eV. This position is shown on the predicted surface phase diagram, Supplementary Fig. 10, with a rectangle representing the relatively large uncertainty in $\Delta\mu_{\mathrm{Ga}}$.

A recent study of reconstructions on GaN (0001) in the OMVPE environment[16] included the effects of additional entropy associated with adsorbed species, which leads to a phase diagram that varies somewhat with temperature, even when expressed in chemical potential coordinates. These effects tend to stabilize reconstructions with H adsorbates at higher T, giving a larger region of phase stability for the 3H(T1) reconstruction than shown in Supplementary Fig. 10. This is consistent with our finding that the 3H(T1) reconstruction agrees best with the experimental CTRs for all conditions studied.

SUPPLEMENTARY DISCUSSION 2: KINK DENSITY

The density of kinks on a step is determined by two terms: the minimum density that is geometrically required to give the average direction of the step, and the additional thermally generated kink pairs[17]. This can be analyzed in terms of the probabilities n_+ and n_- for positive or negative kinks to occur at each lattice site on the step. For a close-packed surface with lattice parameter a, the geometrical requirement gives

$$\theta \equiv n_+ - n_- = 2/(\sqrt{3}/\tan\phi + 1), \tag{9}$$

where ϕ is the angle of the step with respect to the atomic rows in the $[2\bar{1}\bar{1}0]$ type directions. The probabilities must also satisfy

$$n_+ n_- = \exp(-2w/kT), \tag{10}$$

where $2w$ is the energy cost to generate a kink pair, and we assume the kink probabilities are much smaller than unity.

For our sample with $\phi = 5°$ and $a = 3.2$ Å, the geometrically required maximum average kink spacing is $a/\theta = 33$ Å. If we estimate the kink pair energy as $2w = W/6$ where $W = 3.38$ eV is the bulk binding energy per molecule for GaN[18], this gives an average kink spacing of $a/(\theta + 2n_-) = 24$ Å at $T = 1073$ K.

ACKNOWLEDGMENTS

Work supported by the U.S Department of Energy (DOE), Office of Science, Office of Basic Energy Sciences, Materials Science and Engineering Division. Experiments were performed at the Advanced Photon Source beamline 12ID-D, a DOE Office of Science user facility operated by Argonne National Laboratory.

---

* correspondence to: juguangxu@gmail.com; current address: Lumileds Lighting Co., San Jose, CA 95131 USA.
† correspondence to: stephenson@anl.gov

[1] Guangxu Ju, Matthew J. Highland, Angel Yanguas-Gil, Carol Thompson, Jeffrey A. Eastman, Hua Zhou, Sean M. Brennan, G. Brian Stephenson, and Paul H. Fuoss, "An instrument for *in situ* coherent X-ray studies of metal-organic vapor phase epitaxy of III-nitrides," Rev. Sci. Instrum. **88**, 035113 (2017).
[2] Guangxu Ju, Matthew J. Highland, Carol Thompson, Jeffrey A. Eastman, Paul H. Fuoss, Hua Zhou, Roger Dejus, and G. Brian Stephenson, "Characterization of the X-ray coherence properties of an undulator beamline at the Advanced Photon Source," J. of Synchrotron Radiat. **25**, 1036–1047 (2018).
[3] Guangxu Ju, Dongwei Xu, Matthew J. Highland, Carol Thompson, Hua Zhou, Jeffrey A. Eastman, Paul H. Fuoss, Peter Zapol, Hyunjung Kim, and G. Brian Stephenson, "Coherent X-ray spectroscopy reveals the persistence of island arrangements during layer-by-layer growth," Nat. Phys. **15**, 589–594 (2019).
[4] Edith Perret, M. J. Highland, G. B. Stephenson, S. K. Streiffer, P. Zapol, P. H. Fuoss, A. Munkholm, and Carol Thompson, "Real-time x-ray studies of crystal growth modes during metal-organic vapor phase epitaxy of GaN on c- and m-plane single crystals," Appl. Phys. Lett. **105**, 051602 (2014).
[5] G. B. Stephenson, J. A. Eastman, O. Auciello, A. Munkholm, Carol Thompson, P. H. Fuoss, P. Fini, S. P. DenBaars, and J. S. Speck, "Real-time x-ray scattering studies of surface structure during metalorganic chemical vapor deposition of GaN," MRS Bull. **24[1]**, 21 (1999).

[6] D. D. Koleske, M. E. Coltrin, and M. J. Russell, "Using optical reflectance to measure GaN nucleation layer decomposition layer kinetics," J. Cryst. Growth **279**, 37–54 (2005).

[7] J. Tapping and M. L. Reilly, "Index of refraction of sapphire between 24 and 1060 C for wavelengths of 633 and 799 nm," J. Opt. Soc. Am. A **3**, 610–616 (1986).

[8] Y. S. Touloulian, R. K. Kirby, R. E. Taylor, and T. Y. Lee, *Thermal Expansion: Nonmetallic Solids*, Vol. 13 (Springer, 1977) pp. 154–389.

[9] D. D. Koleske, A. E. Wickenden, R. L. Henry, Culbertson. J. C., and M. E. Twigg, "GaN decomposition in H_2 and N_2 at MOVPE temperatures and pressures," J. Cryst. Growth **223**, 466–483 (2001).

[10] Chris G. Van de Walle and J. Neugebauer, "First-principles surface phase diagram for hydrogen on GaN surfaces," Phys. Rev. Lett. **88**, 066103 (2002).

[11] Weronika Walkosz, Peter Zapol, and G. Brian Stephenson, "Metallicity of InN and GaN surfaces exposed to NH_3," Phys. Rev. B **85**, 033308 (2012).

[12] Chris G. Van de Walle and J. Neugebauer, "Role of hydrogen in surface reconstructions and growth of GaN," J. Vac. Sci. Technol. B **20**, 1640–1646 (2002).

[13] Malcolm W. Chase, Jr., "NIST-JANAF thermochemical tables (4th edition)," J. Phys. Chem. Ref. Data, Monograph 9 , 1–1151 (1998).

[14] O. Ambacher, M. S. Brandt, R. Dimitrov, T. Metzger, M. Stutzmann, R. A. Fischer, A. Miehr, A. Bergmaier, and G. Dollinger, "Thermal stability and desorption of Group III nitrides prepared by metal organic chemical vapor deposition," J. Vac. Sci. Technol. B **14**, 3532–3542 (1996).

[15] Fan Jiang, A. Munkholm, R.-V. Wang, S. K. Streiffer, Carol Thompson, P. H. Fuoss, K. Latifi, K. R. Elder, and G. B. Stephenson, "Spontaneous oscillations and waves during chemical vapor deposition of InN," Phys. Rev. Lett. **101**, 086102 (2008), (See online supplemental for trimethylindium (TMI) partial pressures required to condense InN and elemental In).

[16] Pawel Kempisty and Yoshihiro Kangawa, "Evolution of the free energy of the GaN (0001) surface based on first-principles phonon calculations," Phys. Rev. B **100**, 085304 (2019).

[17] W. Burton, N. Cabrera, and F. Frank, "The growth of crystals and the equilibrium structure of their surfaces," Philos. Trans. Royal. Soc. London Ser. A **243**, 299 (1951).

[18] Dongwei Xu, Peter Zapol, G. Brian Stephenson, and Carol Thompson, "Kinetic Monte Carlo simulations of GaN homoepitaxy on c- and m-plane surfaces," J. Chem. Phys. **146**, 144702 (2017).
